# The interplay between autophagy and ROS in tumorigenesis

**DOI:** 10.3389/fonc.2012.00171

**Published:** 2012-11-21

**Authors:** Sameera Kongara, Vassiliki Karantza

**Affiliations:** ^1^Robert Wood Johnson Medical School, University of Medicine and Dentistry of New JerseyPiscataway, NJ, USA; ^2^The Cancer Institute of New JerseyNew Brunswick, NJ, USA; ^3^Division of Medical Oncology, Department of Internal Medicine, Robert Wood Johnson Medical School, University of Medicine and Dentistry of New JerseyPiscataway, NJ, USA

**Keywords:** autophagy, ROS, oxidative stress, cancer, p62, inflammation

## Abstract

Reactive oxygen species (ROS) at physiological levels are important cell signaling molecules. However, aberrantly high ROS are intimately associated with disease and commonly observed in cancer. Mitochondria are primary sources of intracellular ROS, and their maintenance is essential to cellular health. Autophagy, an evolutionarily conserved process whereby cytoplasmic components are delivered to lysosomes for degradation, is responsible for mitochondrial turnover and removal of damaged mitochondria. Impaired autophagy is implicated in many pathological conditions, including neurological disorders, inflammatory bowel disease, diabetes, aging, and cancer. The first reports connecting autophagy to cancer showed that allelic loss of the essential autophagy gene *BECLIN1* (*BECN1*) is prevalent in human breast, ovarian, and prostate cancers and that *Becn1*^+^^/^^-^ mice develop mammary gland hyperplasias, lymphomas, lung and liver tumors. Subsequent studies demonstrated that *Atg5*^-/-^ and *Atg7*^-/-^ livers give rise to adenomas, *Atg4C*^-/-^ mice are susceptible to chemical carcinogenesis, and *Bif1*^-/-^ mice are prone to spontaneous tumors, indicating that autophagy defects promote tumorigenesis. Due to defective mitophagy, autophagy-deficient cells accumulate damaged mitochondria and deregulated ROS levels, which likely contribute to their tumor-initiating capacity. However, the role of autophagy in tumorigenesis is complex, as more recent work also revealed tumor dependence on autophagy: autophagy-competent mutant-Ras-expressing cells form tumors more efficiently than their autophagy-deficient counterparts; similarly, *FIP200* deficiency suppresses PyMT-driven mammary tumorigenesis. These latter findings are attributed to the fact that tumors driven by powerful oncogenes have high metabolic demands catered to by autophagy. In this review, we discuss the relationship between ROS and autophagy and summarize our current knowledge on their functional interactions in tumorigenesis.

## INTRODUCTION

Reactive oxygen species (ROS) are a family of molecules that include highly reactive free oxygen radicals, such as the superoxide anion ($O_{2}^{\cdot -}$) and the hydroxyl radical (OH^.^ ), and stable non-radical oxidants, such as hydrogen peroxide (H_2_O_2_). ROS are produced in cells through metabolism of oxygen and, when at high levels, can function as destructive molecules, actively participating in microbial elimination within phagocytes and also contributing to genomic instability, thereby resulting in cell death and/or tumorigenesis. In more recent years, however, ROS at lower physiological levels have been recognized as intracellular signal transduction molecules that regulate kinase-driven pathways, in turn mediating cellular responses to external stimuli or challenges, such as growth factors, nutrient deprivation, or hypoxia ( Gough and Cotter, 2011). Mitochondria are a major source of intracellular ROS, as leaky electrons from oxidative phosphorylation [1–5% of electrons escape from complexes I and III along the electron transport chain (ETC)] and enzymatic complexes, such as α-glycerophosphate dehydrogenase and α-ketoglutarate dehydrogenase, generate short-lived superoxide anions in the presence of oxygen, which are rapidly converted into relatively stable and membrane diffusible hydrogen peroxide by superoxide dismutase (Mn-SOD), a resident protein of the mitochondrial matrix ( Karihtala and Soini, 2007;  Miao and St Clair, 2009;  Marchi et al., 2012). Other important intracellular sources of ROS include enzymes, such as the nicotinamide adenine dinucleotide phosphate (NADPH) oxidases, (XO), the ER luminal thiol oxidase Ero1, and myeloperoxidase ( Arnhold and Flemmig, 2010;  Tavender and Bulleid, 2010;  Dikalov, 2011;  Fransen et al., 2012), as well as other organelles, such as peroxisomes ( Fransen et al., 2012). Reactive nitrogen species (RNS), such as nitric oxide (NO) and peroxynitrite (ONOO), also act as signaling agents regulating numerous physiological and pathological conditions ( Fukuto et al., 2012;  Lee et al., 2012); however, in this XO, xanthine oxidase. review we will only focus on ROS.

At physiological levels, ROS function as signaling molecules through oxidation of redox-sensitive cysteine residues and resultant inhibition of phosphatases that negatively regulate signaling cascades ( Meng et al., 2002), whereas excessive ROS create an imbalance between the oxidant and antioxidant responses in cells, thus resulting in oxidative stress, which has been etiologically implicated in the pathogenesis of several malignancies, including breast, lung, liver, and prostate cancers ( Moller et al., 2008;  Nowsheen et al., 2009;  Khandrika et al., 2009;  Severi et al., 2010;  Adcock et al., 2011;  Sakaguchi et al., 2011;  Vera-Ramirez et al., 2011;  Thapa and Ghosh, 2012). Abnormally high ROS levels result in oxidation of nuclear and mitochondrial DNA, proteins and lipids, thus impairing their structure and function and causing aberrant expression and regulation of tumor-promoting proteins and/or pathways ( Valko et al., 2006). Indeed, high levels of malondialdehyde, a marker of lipid peroxidation, are observed in plasma samples from patients with breast cancer as compared to normal controls ( Huang et al., 1999;  Gonenc et al., 2001;  Kumaraguruparan et al., 2002;  Polat et al., 2002;  Yeh et al., 2005;  Sener et al., 2007). Conversely, overexpression of the antioxidant enzymes copper/zinc superoxide dismutase (SOD1) and manganese superoxide dismutase (SOD2) in human breast cancer cell lines suppresses xenograft tumor growth ( Weydert et al., 2006), and transgenic overexpression of catalase in PyMT mice diminishes tumor invasiveness and metastasis ( Goh et al., 2011).

Autophagy, derived from the Greek words for *self* (auto) and *eating* (phagy), is an evolutionarily conserved catabolic process that targets proteins, cytoplasm and organelles to lysosomes for degradation, followed by recycling of free amino acids and ATP into the cytoplasm for biomolecule synthesis within the same cell ( He and Klionsky, 2009;  Yang and Klionsky, 2010). Three modes of autophagy exist, namely macroautophagy, microautophagy, and chaperone-mediated autophagy. Microautophagy and chaperone-mediated autophagy are characterized by the direct delivery of cargo into lysosomes, while macroautophagy (hereafter referred to as autophagy) is marked by the formation of double membrane vesicles, which capture cytoplasmic material for delivery to lysosomes. While autophagy occurs at a basal level in most tissue types and promotes cellular homeostasis by recycling proteins and organelles, it is up-regulated in response to cellular insults, such as glucose and amino acid deprivation, hypoxia, oxidative stress, and chemotherapeutic drugs. Impaired autophagy has been implicated in the pathogenesis of diverse diseases, including cancer, diabetes, Crohn’s disease, and neurodegenerative conditions, like Alzheimer’s, Parkinson’s, and Huntington’s disease ( Sridhar et al., 2012;  Quan et al., 2012).

The role of autophagy in tumorigenesis is complex. Allelic loss of the essential autophagy regulator *BECLIN1* (*BECN1*) is observed in over 50% of human breast, ovarian, and prostate cancers ( Aita et al., 1999), and *Becn1*^+^^/^^-^ mice are susceptible to hepatocellular carcinomas, lung adenocarcinomas, lymphomas, and mammary gland hyperplasias ( Qu et al., 2003;  Yue et al., 2003). Furthermore, autophagy-compromised, apoptosis-defective immortalized mouse mammary epithelial cells (iMMECs) and baby mouse kidney (iBMK) cells generate allograft tumors in nude mice more efficiently than their wild-type counterparts ( Karantza-Wadsworth et al., 2007;  Mathew et al., 2007). Mice lacking *Bif1*, a positive regulator of autophagy, are prone to spontaneous lymphomas, sarcomas, duodenal adenocarcinomas, small cell lung carcinomas, esophageal squamous cell carcinomas, and hepatocellular carcinomas ( Takahashi et al., 2007), whereas *Atg4C*^-/-^ mice are susceptible to chemical-induced fibrosarcomas ( Marino et al., 2007), and *Atg5* or *Atg7* deletion in the liver results in hepatocellular adenomas ( Takamura et al., 2011;  Inami et al., 2011). Paradoxically though, autophagy is required for the robust progression of tumors ( Guo et al., 2011;  Lock et al., 2011;  Kim et al., 2011;  Yang et al., 2011) and also supports PyMT-driven mammary tumorigenesis, as loss of *FIP200*, a positive regulator of autophagy, inhibits primary tumor formation and metastasis ( Wei et al., 2011). This conundrum can be, at least partially, attributed to the important roles of autophagy in both maintenance of cellular homeostasis under regular growth conditions and protection of cell viability under stress. As a housekeeping pathway, autophagy degrades damaged or aggregated proteins and organelles, which are known sources of ROS, thereby preventing tumor initiation via suppression of oxidative and genotoxic stress ( Karantza-Wadsworth et al., 2007;  Mathew et al., 2007). On the other hand, oncogene activation increases the metabolic demands of transformed cells and intact autophagy supports tumor cell survival by providing substrates for mitochondrial metabolism ( Guo et al., 2011). How the functional status of autophagy impacts tumorigenesis is still under investigation, and tissue- and context-specific roles are likely to be revealed.

This review will focus on the interplay between ROS and autophagy and their functional interactions in the context of cancer.

## AUTOPHAGIC MACHINERY

Early work led to the identification of several *Atg* (*A*u*t*opha*g*y-related) genes, which are indispensable for the functional execution of the autophagic cascade in yeast. Most yeast Atg proteins have mammalian homologs participating in a similarly ordered process. Initiation of autophagy is characterized by the formation of nascent double membrane structures called phagophores, which elongate and enclose cytoplasmic material in vesicles or autophagosomes. Mature autophagosomes subsequently fuse with lysosomes to form autolysosomes, thereby leading to the degradation of cargo and the release of amino acids and ATP into the cytoplasm ( He and Klionsky, 2009;  Yang and Klionsky, 2010).

In mammalian cells, under nutrient-rich conditions, the nutrient sensor and serine/threonine kinase mammalian target of rapamycin complex 1 (mTORC1) phosphorylates ULK1/2, and mAtg13, preventing the assembly of a multi-protein complex, which is required for autophagosome formation. Under starvation, mTORC1 is inactivated, ULK1/2 undergo autophosphorylation, and then phosphorylate mAtg13 and FIP200 to form an ULK1/2–mAtg13–FIP200 complex that is stabilized by Atg10 ( Hara et al., 2008;  Ganley et al., 2009;  Hosokawa et al., 2009;  Jung et al., 2009;  Mercer et al., 2009). The nucleation and formation of the phagophore also requires the Class III PI3K/Vps34 complex, which consists of PI3K/Vps34, the serine/threonine kinase p150 and Beclin1; this complex is positively regulated by Atg14L, the ultraviolet irradiation resistant-associated gene (UVRAG), the Bax-interacting factor 1 (Bif-1), and the activating molecule in Beclin 1-regulated autophagy (AMBRA), and negatively regulated by the RUN domain Beclin 1-interacting cysteine-rich-containing protein (RUBICON;  Kihara et al., 2001;  Liang et al., 2006;  Fimia et al., 2007;  Takahashi et al., 2007;  Sun et al., 2008; reviewed in  Yang and Klionsky, 2010). The ULK and the Class III PI3K complexes recruit two ubiquitin-like conjugation systems for expansion of the autophagosome membrane. Following activation by Atg7 (E1-like enzyme), Atg12 is transferred to Atg10 (E2-like enzyme) and becomes covalently bound to Atg5 via a lysine residue. Atg16L combines with Atg5–Atg12 to form the Atg5–Atg12–Atg16L complex that self-oligomerizes and binds to the phagophore ( Mizushima et al., 1998,  2001,  2003;  Tanida et al., 2001; reviewed in  He and Klionsky, 2009). Similarly, following cleavage at its C-terminus by the cysteine protease Atg4 and activation by Atg7 (E1-like enzyme), Atg8 (or LC3) is transferred to Atg3 (E2-like enzyme), conjugated to phosphatidylethanolamine (PE) and incorporated into the autophagosome membrane, in a process facilitated by – but not exclusively dependent on – the Atg5–Atg12 complex ( Kabeya et al., 2000;  Tanida et al., 2002;  Nishida et al., 2009; reviewed in  He and Klionsky, 2009). Atg4 can also delipidate LC3-PE to release LC3 from autophagosomes and, thus, increase the free LC3 pool ( Kabeya et al., 2004;  Tanida et al., 2004). The protein p62/SQSTM1, which accumulates under conditions of oxidative stress, binds to ubiquitinated proteins via its ubiquitin-associated (UBA) domain, sequesters them by self-oligomerization, and delivers them to autophagosomes via its LC3 binding domain ( Bjorkoy et al., 2005;  Komatsu et al., 2007;  Pankiv et al., 2007). Finally, the autophagosomal cargo, including p62, is degraded in lysosomes ( Bjorkoy et al., 2005;  Komatsu et al., 2007;  Pankiv et al., 2007).

## CROSSTALK BETWEEN ROS AND AUTOPHAGY: ROS MODULATE AUTOPHAGY

### AUTOPHAGY INDUCTION IN RESPONSE TO ROS

#### Direct effect on Atg proteins

Amino acid deprivation induces the formation of H_2_O_2_ in mitochondria in a Class III PI3K-dependent manner, and this ROS is essential for the induction of autophagy in response to starvation ( Scherz-Shouval et al., 2007). Specifically, the Cys81 residue near the catalytic site of Atg4 is a direct oxidation target by H_2_O_2_; its oxidized form inactivates the protease activity of Atg4 and prevents the delipidation of LC3 without affecting the C-terminal processing of LC3 by Atg4, thus leading to increased autophagosome formation. In a reducing environment, the Atg4 protease remains active and delipidates LC3, thereby suppressing autophagosome membrane formation and resulting in autophagy inhibition.

#### Indirect induction of autophagy

The energy-dependent activity of mTOR is regulated by the heterotrimeric enzyme AMPK, which is in turn activated by phosphorylation in response to high intracellular AMP/ATP ratios. Under nutrient-depleted conditions, active AMPK phosphorylates TSC2, as well as the mTORC1 subunit Raptor, effectively preventing the activation of mTORC1 and, therefore, inducing autophagy ( Inoki et al., 2003;  Gwinn et al., 2008). More recent studies demonstrate a direct role for AMPK in the induction of autophagy. Under glucose deprivation, AMPK directly activates ULK1 by phosphorylation at Ser317 and Ser777 and results in the induction of autophagy ( Kim et al., 2011). In another study, phosphorylated ULK1 (at Ser555) is detected in wild-type hepatocytes, but not in hepatocytes from *AMPK*^-/-^ mice treated with metformin, an activator of AMPK ( Egan et al., 2011). Reconstitution of ULK1 in *ULK1*^-/-^ MEFs, but not a mutated ULK1 construct lacking the AMPK phosphorylation site, rescues the viability of cells under starvation by the induction of mitochondrial-specific autophagy ( Egan et al., 2011). AMPK is a major mediator of indirect activation of autophagy by ROS, as it is sensitive to oxidative stress induced by H_2_O_2_ and gets phosphorylated at Thr172 of its α1 catalytic subunit ( Choi et al., 2001). AMPK also induces autophagy in Atg5- and LKB1-, but not AMP/ATP ratio-, dependent manners in response to hypoxia-associated mitochondrial ROS production ( Papandreou et al., 2008;  Emerling et al., 2009).

Reactive oxygen species-associated autophagy induction is also mediated by ataxia-telangiectasia mutated (ATM), a kinase that is critical for orchestrating cellular responses to DNA damage and, in parallel to its nuclear functions, increases TSC2 activity in the cytoplasm via the LKB1/AMPK metabolic pathway, thus resulting in mTORC1 inactivation ( Alexander et al., 2010). Similarly, ROS-induced and p53-activated sestrins (Sesns) have been shown to suppress oxidative stress by promoting autophagy via the AMPK-mTOR axis in *Drosophila* ( Lee et al., 2010;  Budanov, 2011)

High mobility group box 1 (HMGB1) is a nuclear protein that is released extracellularly in response to cytokines, trauma, and cell death, and can mediate inflammation by binding to receptors, such as the receptor for advanced glycation end products (RAGE) and Toll-like receptors (TLRs;  Lotze and Tracey, 2005;  Sims et al., 2010). Recent studies demonstrated that loss of HMGB1 attenuates autophagy ( Tang et al., 2010a,b). Furthermore, HMGB1 translocates from the nucleus to the cytoplasm in response to autophagy-inducing stimuli in a ROS-dependent manner, since treatment with the antioxidant *N*-acetyl cysteine (NAC) dampens, while loss of the antioxidant enzymes SOD1 and SOD2 promotes, autophagy induction and HMGB1 cytoplasmic translocation ( Tang et al., 2010b). HMGB1, in turn, promotes autophagy by disrupting the Beclin1–Bcl-2 interaction and enhancing ERK1/2 activity ( Tang et al., 2010b). The redox sensitivity of HMGB1 is critical for autophagy induction, as oxidation of cysteine residues at positions 23 and 45 is essential for the ability of HMGB1 to disrupt the Beclin1–Bcl-2 interaction, while Cysteine 106 is important for the cytoplasmic localization of HMGB1 ( Tang et al., 2010b). Similarly, the HMGB1 receptor RAGE positively regulates autophagy and promotes survival of pancreatic cancer cells upon treatment with hydrogen peroxide ( Kang et al., 2011a,b).

Hypoxia triggers an acute induction of ROS over a short period of time, while prolonged hypoxia results in decreased intracellular ROS levels as compared to normoxia ( Semenza, 2011). Mitochondrial ROS generated in response to hypoxia prevent degradation of the hypoxia-inducible factor-1 alpha (HIF-1α;  Chandel et al., 2000;  Guzy et al., 2005). Hypoxia-induced stabilization of HIF-1α leads to transcription of several target genes, including BNIP3 and BNIP3L, which induce autophagy in response to low oxygen conditions ( Bruick, 2000;  Sowter et al., 2001;  Zhang et al., 2008;  Bellot et al., 2009). In addition to hypoxia, BNIP3 mediates autophagy induction in response to H_2_O_2_ and during myocardial ischemia/reperfusion injury ( Hamacher-Brady et al., 2007;  Byun et al., 2009). H_2_O_2_-responsive BNIP3-mediated induction of autophagy occurs via dephosphorylation of mTOR at the S2481 residue, resulting in its inactivation ( Byun et al., 2009), while hypoxia-driven autophagy induction relies on the ability of BNIP3 to bind to and interfere with the activity of the GTPase Rheb, a positive regulator of mTORC1, thereby preventing TORC1 activation ( Li et al., 2007). Additionally, mutating a redox-sensitive cysteine residue at position 64 diminishes the ability of BNIP3 to undergo homodimerization in response to oxidative stress, such as that caused by ischemia and reperfusion ( Kubli et al., 2008), and this homodimerization is critical for the interaction of BNIP3 with LC3 and the induction of autophagy ( Hanna et al., 2012).

Other mediators of autophagy induction in response to ROS and oxidative stress include the forkhead transcriptional regulator FOXO1 and FOXO3 ( Sengupta et al., 2011), the p38 mitogen-activated protein kinase (MAPK;  Cheng et al., 2009;  McClung et al., 2010), the extracellular regulated kinase (ERK) and the c-Jun N-terminal kinase (JNK;  Wu et al., 2009;  Wong et al., 2010), and the endoplasmic reticulum kinase PERK ( Avivar-Valderas et al., 2011).

### INHIBITORY EFFECT OF ROS ON POSITIVE REGULATORS OF AUTOPHAGY

#### Direct effect on Atg proteins

Another point of direct autophagy regulation by ROS may be at the two ubiquitin-like conjugation systems described earlier. While direct evidence is lacking in this regard, it has been speculated that the sulfhydryl group-containing ubiquitin-like enzymes Atg3, Atg7, and Atg10 are redox-sensitive in a manner similar to the E1 and E2 enzymes of the ubiquitin system, which possess catalytic cysteines and can undergo reversible oxidation and inactivation in the presence of ROS ( Filomeni et al., 2010).

#### Indirect effect on other positive autophagy regulators

Reactive oxygen species can also modulate autophagy indirectly by affecting the activity of the master autophagy regulator mTORC1 rather than the Atg proteins themselves. mTORC1, a multi-protein complex consisting of the conserved serine/threonine kinase mTOR, Raptor, mLST8, PRAS40, and Deptor, is sensitive to the availability of amino acids, growth factors, hormones, and energy, and subsequently regulates translation, cell growth, and autophagy ( Zoncu et al., 2011). The GTPases Rheb and Rag regulate the activity and the spatial localization of mTORC1, respectively, in response to amino acid and growth factor availability. Rheb is in turn regulated by the GTP activating proteins (GAP) TSC1/TSC2: upon amino acid and growth factor stimulation, PKB/Akt inactivates TSC2 by phosphorylation, resulting in Rheb and mTORC1 activation and concomitant suppression of autophagy ( Zoncu et al., 2011). An earlier study indicated that mTORC1 activity increases in the presence of oxidizing agents, such as diamide or the cysteine oxidant phenylarsine oxide (PAO;  Sarbassov and Sabatini, 2005), and more recent work demonstrated that PAO activates mTORC1 via Rheb ( Yoshida et al., 2011). Interestingly, *Tsc1*^-/-^ and *Tsc2*^-/-^ MEFs exhibit constitutively high, rapamycin-sensitive mTOR activity regardless of redox modulation by an oxidant (PAO) or a reducing (British anti-Lewisite) agent, thus indicating that TSC1/2 maybe redox-sensitive proteins and potentially regulated by ROS ( Yoshida et al., 2011).

mTOR activation due to growth factor stimulation is mediated by a number of signaling pathways, including the PI3K–Akt axis. For instance, binding of insulin to its receptor recruits PI3K to the cell membrane, where it phosphorylates phosphatidylinositol-4,5-phosphate (PIP_2_) to generate phosphatidylinositol-3,4,5-phosphate (PIP_3_) in a reaction that is reversibly catalyzed by the phosphatase-and-PI3K antagonist PTEN ( Hernandez-Aya and Gonzalez-Angulo, 2011;  Zoncu et al., 2011). Formation of PIP_3_ leads to recruitment and activation of PH-domain proteins, such as PDK1 and PKB/Akt, leading to further downstream signaling and mTORC1 activation. Activation of the PI3K axis in response to growth factors, such as EGF, insulin, and PDGF, is facilitated by concomitant production of H_2_O_2_, which inactivates PTEN by oxidizing a cysteine residue (Cys124) at its catalytic site and reversibly cross-linking it to Cys71 via a disulfide bond ( Lee et al., 2002;  Kwon et al., 2004). Although not formally demonstrated, ROS-induced inactivation of PTEN can result in increased mTORC1 activity via the PI3K–Akt pathway, thus leading to suppression of autophagy. An antioxidant buffer ameliorating growth factor-associated ROS production, and thus preserving autophagy in the context of PI3K axis activation, is provided by peroxiredoxins, particularly peroxiredoxin I (Prdx1) and peroxiredoxin II (Prdx2), which modulate PTEN oxidation ( Kwon et al., 2004;  Cao et al., 2009). Interestingly, MMTV-v-*H-Ras*;*Prdx1*^-/-^ mice develop mammary tumors at a higher frequency compared to MMTV-v-*H-Ras*;*Prdx1*^+^^/^^+^ mice, possibly in association with increased PTEN oxidation and resultant PI3K/Akt/mTOR axis activation, as mammary epithelial cells isolated from MMTV-v-*H-Ras*; *Prdx1*^-/-^ mice demonstrate higher levels of oxidized PTEN compared to their wild-type counterparts ( Cao et al., 2009). These studies indicate that ROS may negatively regulate autophagy by mTORC1 induction via oxidation and inactivation of PTEN and TSC1/2, and suggest that other mechanism(s) protective against mTORC1 activation must be simultaneously active, so that autophagy is allowed to proceed as a prosurvival cell function under oxidative stress.

## CROSSTALK BETWEEN ROS AND AUTOPHAGY: AUTOPHAGY MODULATES MITOCHONDRIAL ROS

While the discussion has thus far focused on the impact of ROS on the functional status of autophagy in response to various cellular stressors, the autophagic pathway in turn modulates the cellular levels of ROS. Autophagy is essential for the turnover of normal mitochondria, as well as the removal of damaged mitochondria, which are primary sources of intracellular ROS. This specific form of autophagy is called mitophagy ( Lemasters, 2005;  Youle and Narendra, 2011). Autophagy-deficient cells have significantly higher ROS levels compared to their wild-type counterparts ( Mathew et al., 2009;  Kongara et al., 2010). The ROS scavenger NAC improves autophagy-deficient cell survival under metabolic stress in association with attenuation of p62 accumulation, indicating that excessive ROS results in p62 up-regulation and impairment of cell survival ( Mathew et al., 2009;  Kongara et al., 2010). Mammary tumors from *FIP200*^f/f^;MMTV-*Cre*;MMTV-*PyMT* mice are characterized by the presence of abnormal mitochondria, as well as accumulation of both healthy and dysfunctional mitochondria compared to the wild-type controls ( Wei et al., 2011), and conditional deletion of *FIP200* in hematopoietic stem cells and neural cells results in mitochondria accumulation and increased ROS levels ( Liang et al., 2010;  Liu et al., 2010). Morphologically abnormal mitochondria are also observed in *Atg7*^-/-^ livers ( Komatsu et al., 2005;  Inami et al., 2011) and pancreatic β cells ( Ebato et al., 2008;  Jung et al., 2008), *Atg5*^-/-^ cardiac tissue ( Nakai et al., 2007), and Paneth cells with a hypomorphic allele of *Atg16L1* ( Cadwell et al., 2008). Furthermore, increased ROS levels are associated with increased mitochondrial content in *Atg5*^-/-^ MEFs ( Tal et al., 2009) and *Atg7*^-/-^ T cells ( Pua et al., 2009). Taken together, all these studies indicate that mitophagy plays an essential role in recycling of functional and removal of damaged mitochondria and clearly demonstrate that defective autophagy-associated blocks in mitophagy lead to increased intracellular levels of ROS, which in turn may be etiologically linked to tumorigenesis.

Mitophagy has been extensively examined in normal development, such as erythrocyte maturation, which involves developmental elimination of mitochondria from red blood cells, as well as in the context of mitochondrial depolarization induced by chemicals, such as CCCP (carbonyl cyanide m-chlorophenylhydrazone). Mitochondrial elimination during erythrocyte maturation depends upon the outer mitochondrial membrane protein NIP3-like protein X (Nix, also known as BNIP3L), since *Nix*^-/-^ reticulocytes retain their mitochondria ( Schweers et al., 2007;  Sandoval et al., 2008). Similarly, *ULK1*^-/-^ and *Atg7*^-/-^ reticulocytes exhibit delayed mitochondrial clearance ( Kundu et al., 2008;  Zhang et al., 2009). *Atg7*^-/-^ reticulocytes also accumulate damaged mitochondria, accompanied by increased ROS ( Mortensen et al., 2010). Although Nix has been shown to directly interact with LC3 via its WXXL motif ( Novak et al., 2010), its precise role in mitochondrial clearance remains unclear.

While Nix is involved in removal of functional mitochondria during normal red blood cell development, the serine/threonine kinase PTEN-induced kinase1 (PINK1) and the E3 ubiquitin ligase Parkin are essential for the elimination of damaged or dysfunctional mitochondria. PINK1 is constitutively synthesized and proteolytically degraded in normal mitochondria. Loss of membrane potential and depolarization of damaged mitochondria results in stabilization and accumulation of PINK1, which in turn recruits cytoplasmic Parkin to abnormal mitochondria and triggers mitophagy ( Matsuda et al., 2010;  Narendra et al., 2008,  2010a;  Vives-Bauza et al., 2010). Parkin ubiquitinates mitochondrial proteins involved in mitochondrial fusion, such as mitofuscin 1 and 2 (MFN1 and 2), and results in their degradation ( Gegg et al., 2010;  Poole et al., 2010;  Rakovic et al., 2011). The precise function of this degradation remains unknown, as are the details of how Parkin stimulates mitophagy. p62/SQSTM1 is recruited to damaged mitochondria by Parkin, but there are conflicting reports regarding its requirement for mitophagy. Reduced mitochondrial clearance is observed in HeLa cells with a transient p62 knockdown upon treatment with the mitochondrial uncoupler CCCP ( Geisler et al., 2010). On the other hand, *p62*^-/-^ MEFs do not have a defect in mitochondrial clearance upon CCCP treatment, despite the finding that p62 causes perinuclear, and thus autophagosome-like, mitochondrial clustering ( Okatsu et al., 2010;  Narendra et al., 2010b).

## FUNCTIONAL INTERACTIONS BETWEEN AUTOPHAGY AND ROS INTUMORIGENESIS

### DNA DAMAGE AND GENOMIC INSTABILITY

As mentioned above, *Becn1*^+^^/^^-^ iMMECs ( Kongara et al., 2010) and iBMK cells ( Mathew et al., 2009) exhibit elevated intracellular ROS levels compared to their wild-type counterparts and, although not directly demonstrated, oxidative stress due to excess ROS is likely contributing to the DNA damage and genomic instability observed in autophagy-defective iMMECs and iBMK cells *in vitro* and in their resultant allograft tumors in nude mice *in vivo* ( Karantza-Wadsworth et al., 2007;  Mathew et al., 2007;  Mathew et al., 2009), thus providing a plausible link between autophagy defects, ROS accumulation, and enhanced tumorigenic potential, especially in the background of impaired apoptosis and cell cycle check-point defects ( Karantza-Wadsworth et al., 2007).

### ROLE OF p62

Loss of *Atg5* and *Atg7* in mouse livers results in accumulation of abnormal mitochondria, formation of inclusion bodies, liver injury, and development of benign hepatocellular adenomas ( Komatsu et al., 2007;  Inami et al., 2011;  Takamura et al., 2011). p62 is a component of these inclusion bodies, which are cytoplasmic aggregates of ubiquitinated proteins commonly associated with pathological conditions, such as Alzheimer’s ( Kuusisto et al., 2002), Huntington’s ( Nagaoka et al., 2004), and Parkinson’s ( Kuusisto et al., 2001) diseases, as well as alcoholic and non-alcoholic steatohepatitis ( Zatloukal et al., 2002) and hepatocellular carcinoma ( Zatloukal et al., 2002). Combined loss of p62 and autophagy in *Atg7*^-/-^; *p62*^-/-^ livers abrogates inclusion body formation, alleviates hepatic injury and retards tumor progression compared to *Atg7*^-/-^ deficiency alone ( Komatsu et al., 2007;  Takamura et al., 2011). On the contrary, p62 overexpression increases ROS and accelerates tumorigenesis in an autophagy-defective background, but not in an autophagy-competent one ( Mathew et al., 2009), suggesting that the tumor-forming abilities of autophagy-deficient cells may be dependent on persistent and abnormal p62 accumulation, which is the result of combined p62 induction in response to defective autophagy-associated oxidative stress and impaired p62 elimination, again due to deficient autophagy. As explained below, p62 in turn activates prosurvival antioxidant cell responses, including the nuclear factor erythroid 2-related factor 2 (Nrf2) pathway, but in aggregate form also leads to further ROS production, thereby maintaining a potentially tumorigenic vicious cycle.

p62/SQSTM1 activates the Nrf2 pathway in a non-canonical manner and likely modulates ROS levels as a consequence. Nrf2 is a transcription factor, which under normal growth conditions is constitutively targeted for degradation by the Kelch-like ECH-associated protein 1 (Keap1) complex ( Taguchi et al., 2011). Upon induction of oxidative stress, Keap1 undergoes conformational changes leading to the release, stabilization, and translocation of Nrf2 into the nucleus, where it activates the transcription of genes under control of the antioxidant responsive element (ARE), which in turn function to restore physiological ROS levels ( Taguchi et al., 2011). p62 competes with Nrf2 for binding sites on Keap1, resulting in Nrf2 stabilization and enabling its nuclear translocation and activation of target genes, including p62, thus resulting in a positive feedback loop ( Jain et al., 2010;  Komatsu et al., 2010;  Lau et al., 2010). Indeed, under autophagy-deficient conditions, Keap1 is a component of inclusion bodies together with p62 and is, similar to p62, also degraded via autophagy ( Jain et al., 2010;  Komatsu et al., 2010). As expected, liver-specific *Atg7* loss activates the Nrf2 pathway, while simultaneous *p62* loss abrogates this activation ( Komatsu et al., 2007;  Inami et al., 2011). Importantly, similar to concurrent *p62* and *Atg7* loss, combined *Nrf2* and *Atg7* deficiency alleviates the cellular injury observed in *Atg7*^-/-^ livers, whereas combined loss of *Keap1* and *Atg7* exacerbates hepatocyte damage ( Komatsu et al., 2010). Taken together, these results indicate that liver injury due to autophagy defects is driven by p62 accumulation and its resultant activation of the Nrf2 pathway. In support of this hypothesis, *p62* deletion in the human hepatocellular carcinoma cell line Jhh-5 diminishes its colony-forming ability in soft agar in conjunction with failure to activate Nrf2 ( Inami et al., 2011). Whether Nrf2 activity is required for the formation of tumors in the context of autophagy deficiency and p62 accumulation *in vivo* remains to be investigated.

In addition to up-regulating the Nrf2 antioxidant response, p62 modulates ROS levels by also activating the nuclear factor kappa B (NF-κB), which is known to be induced by and also regulate ROS ( Duran et al., 2008;  Morgan and Liu, 2011). The absence of p62 delays tumor formation and reduces tumor burden in a tetracycline-inducible K-Ras model of lung tumorigenesis, and this effect is attributed to loss of NF-κB activity in the *p62*^-/-^ background ( Duran et al., 2008). Similarly, H-Ras^V12^-transformed *p62*^-/-^ MEFs, when compared to their wild-type counterparts, exhibit higher levels of apoptosis in association with increased JNK activity, which in turn is caused by elevated ROS levels ( Duran et al., 2008). Treatment of H-Ras^V12^-transformed *p62*^-/-^ MEFs with the antioxidant butylated hydroxyanisole (BHA) restores cell viability, indicating that excessive ROS is responsible for the increase in cell death ( Duran et al., 2008). Intriguingly, in an autophagy-deficient background, p62 aggregation and oligomerization impair its ability to activate the NF-κB pathway, thereby mimicking a functional loss of p62 with respect to this particular pathway, and may partly explain the increased ROS associated with autophagy deficiency ( Mathew et al., 2009). Thus, p62 accumulation accompanying impaired autophagy fails to activate the NF-κB pathway, leading to increased ROS production, while it simultaneously activates the antioxidant Nrf2 pathway via Keap1 sequestration. The balance between increased ROS production and ROS tempering via the Nrf2 pathway may be an important player in tumor progression in the context of autophagy deficiency.

### INFLAMMASOME ACTIVATION

Inflammasomes are multi-protein complexes responsible for the processing of caspase-1 and the subsequent maturation and secretion of the proinflammatory cytokines interleukin IL-1β and IL-18 ( Martinon et al., 2009). The nucleotide-binding oligomerization domain (NOD)-like receptor (NLR) family, pyrin domain-containing 3 (NLRP3) inflammasome consists of the NLR family member NLRP3, the adaptor apoptosis-associated speck-like (ASC) protein and pro-caspase-1, and is activated in response to diverse stimuli, such as bacteria, viruses, silica, asbestos, uric acid crystals, ATP, lysosomal damage, and impaired phagocytosis ( Martinon et al., 2009;  Gross et al., 2011). Proteolytic activity of the NLRP3 inflammasome requires two steps: (1) priming, which is characterized by the transcriptional up-regulation of NLRP3 and pro-IL-1β, and (2) activation, which involves the oligomerization of NLRP3 in response to various stimuli ( Chen and Nunez, 2011;  Gross et al., 2011). Interestingly, ROS are critical mediators of inflammasome activity, most likely at the priming step ( Bauernfeind et al., 2011), since antioxidants attenuate IL-1β maturation in response to uric acid crystals ( Petrilli et al., 2007;  Dostert et al., 2008), asbestos ( Dostert et al., 2008), and ATP ( Cruz et al., 2007;  Dostert et al., 2008). In agreement with the above findings, defective autophagy-induced mitochondrial ROS also lead to inflammasome activation ( Nakahira et al., 2011;  Zhou et al., 2011), and macrophages isolated from *Atg7*^-/-^, *Atg16L1*^-/-^, *LC3B*^-/-^, and *Becn1*^+^^/^^-^ mice secrete elevated levels of IL-1β and IL-18 upon stimulation ( Saitoh et al., 2008;  Nakahira et al., 2011), thus intimately linking autophagy impairment to inflammasome activation and its resultant inflammatory response.

The connection between inflammasome-associated cytokine production and tumorigenesis is complex, as IL-1β and IL-18 have both pro- and anti-tumorigenic effects. In support of a tumor-promoting role, loss of IL-1β slows the progression of tumors in a model of chemical-induced carcinogenesis ( Krelin et al., 2007), implantation of B16 melanoma cells in *IL-1β*-null mice prevents the formation of tumors as well as lung metastases ( Vidal-Vanaclocha et al., 2000;  Voronov et al., 2003), Lewis lung carcinoma cells overexpressing IL-1β exhibit accelerated rate of tumor formation accompanied by increased vasculature ( Saijo et al., 2002), and IL-1β-overexpressing A459 cells form lung metastasis more efficiently than control cells, in a process inhibited by an antibody to IL-1β ( Yano et al., 2003). Furthermore, implantation of mammary carcinoma 4T1 cells in *IL-1* receptor-null (*IL-1R*^-/-^) mice slows tumor progression in association with immune suppression ( Bunt et al., 2007), and fibroblast growth factor receptor-1 (FGFR-1)-mediated mammary tumorigenesis is dependent on the induction of IL-1β and inhibited by administration of anti-IL-1β antibody ( Reed et al., 2009). Taken together, these studies implicate IL-1β in promotion of tumor cell proliferation, migration, angiogenesis, invasion, and inhibition of immune surveillance. Studies conducted on IL-18 indicate that IL-18 increases apoptosis and decreases metastasis in experimental models of bone metastasis ( Ohtsuki et al., 1997;  Nakata et al., 1999;  Iwasaki et al., 2002); however, IL-18 has also been reported to increase angiogenesis ( Park et al., 2001) and suppress immune surveillance ( Terme et al., 2011).

While earlier studies indicated that IL-1β and IL-18, together with other components of the inflammasome, exacerbate colitis and promote colitis-associated cancer ( Siegmund et al., 2001a,b;  Sivakumar et al., 2002;  Ishikura et al., 2003;  Loher et al., 2004;  Bauer et al., 2007), more recent evidence suggests that these molecules play a protective role by impeding colitis and progression to cancer ( Takagi et al., 2003;  Allen et al., 2010;  Dupaul-Chicoine et al., 2010;  Salcedo et al., 2010;  Zaki et al., 2010a,b). The reason for this discrepancy is unclear, although it can be partly attributed to differences in gut-residing microflora and in experimental design ( Zaki et al., 2011). Furthermore, components of the inflammasome, and in particular IL-1β, are required for the efficient recruitment of the immune system to attack tumor cells upon chemotherapeutic treatment ( Ghiringhelli et al., 2009). In conclusion, modulation of tumor growth and survival by IL-1β and IL-18 is complex and likely context-dependent. Similarly, how inflammasome activation due to autophagy defects impacts tumorigenesis remains unclear and has understandably become a vigorously investigated subject.

## Summary

**FIGURE 1:**
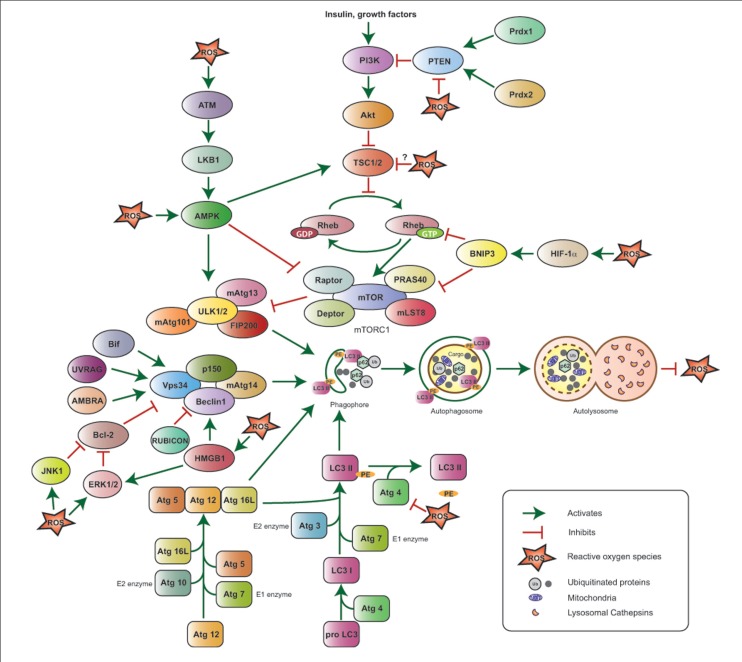
**Reactive oxygen species-mediated autophagy regulation**. The ULK1/2–FIP200–mAtg13 and Beclin1–Vps34–p150 protein complexes together with the two ubiquitin-like proteins Atg12 and LC3 (or Atg8) and their conjugating proteins, including Atg7 (E1-like enzyme), Atg10 (E2-like enzyme), and Atg3 (E2-like enzyme), are the primary regulators of autophagosome formation and, thus, autophagy induction. Atg4 cleaves Pro-LC3 at its C-terminal-end, Atg7 and Atg3 add phosphatidyl ethanolamine (PE), and LC3-PE is then incorporated into the autophagosome membrane; Atg4 can also cleave LC3-PE to release free LC3 and negatively impact autophagy. The PI3K–Akt–mTORC1 pathway suppresses autophagy by inhibiting the ULK1/2–FIP200–mAtg13 complex. PI3K is activated in response to hormones and growth factors and suppresses TSC1/2 activity, thus resulting in Akt and mTORC1 activation; the phosphatase PTEN antagonizes PI3K, while the peroxiredoxins Prdx1 and 2 maintain PTEN function by preventing its oxidation. mTORC1 is also negatively regulated by HIF-1α via BNIP3 induction and by ATM via LKB1 and AMPK. The Beclin1–Vps34–p150 complex is positively regulated by Bif-1, UVRAG, AMBRA, and HMGB1, and negatively regulated by Bcl-2 and RUBICON. ROS stimulate autophagy by activating HIF-1α, AMPK, ATM, HMGB1, JNK1, ERK1/2 and inhibiting the PE-cleaving activity of Atg4, without affecting its ability to process the C-terminal end of LC3. ROS inhibit PTEN and are hypothesized to negatively affect TSC1/2 activity, potentially inhibiting autophagy. Upon its induction, autophagy results in ROS suppression.

**FIGURE 2:**
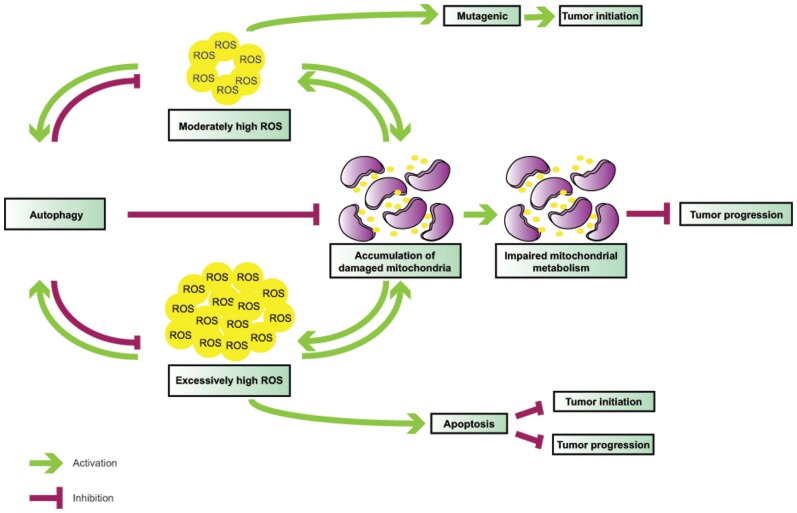
**Interplay between ROS and autophagy in tumorigenesis**. Autophagy is induced by ROS and functions to mitigate oxidative stress. Defective autophagy leads to ROS accumulation and mitochondrial damage; in turn, malfunctioning mitochondria become additional sources of ROS, thereby creating a vicious cycle. While moderately high ROS levels are mutagenic and likely mediate tumor initiation in association with autophagy defects, very high ROS levels may result in apoptosis, and, thus, inhibit both tumor initiation and progression.

The functional interactions between autophagy and ROS are elaborate (**Figure 1**) and their role in tumorigenesis is complex (**Figure 2**), and likely tissue- and context-dependent. Excess ROS up-regulate autophagy by multiple and at times counteracting mechanisms, and in turn this catabolic cellular process restores physiological ROS levels. Defective autophagy promotes the accumulation of damaged mitochondria and results in oxidative stress, which may be partly responsible for the tumor-forming abilities of autophagy-deficient cells by functioning as a genotoxic and mutagenic agent and by triggering secondary, non-cell autonomous effects, such as inflammation.

The finding that *Atg5*^-/-^ or *Atg7*^-/-^ mouse livers give rise to benign adenomas, but not malignant hepatocellular carcinomas, is likely indicative of the opposing roles that autophagy may plays at different stages of tumorigenesis. Chronic impairment of autophagy may be detrimental to cellular health due to accumulation of aggregated proteins and damaged organelles, such as aging mitochondria, thus leading to steadily rising, protumorigenic levels of ROS over time. Since autophagy is one of the primary mechanisms by which cells moderate ROS, autophagy impairment can create a vicious cycle, where inability to temper slightly elevated ROS levels leads to further ROS production, which, if it exceeds a critical threshold, may trigger cell death, and thus, impede tumor progression. Accumulating evidence indicates that tumors driven by metabolically demanding oncogenes, such as mutant Ras and PyMT, also depend on autophagy. Indeed, oncogenic Ras-driven tumors rely on mitochondrial oxidative phosphorylation to meet their metabolic needs, and autophagy is critical for the supply of essential metabolic intermediates and the maintenance of mitochondrial health. In parallel to increased metabolic demands, potent oncogene activation is also associated with excess ROS production and, thus, autophagy may not only support cancer metabolism, but also function as a toxic ROS scavenger to again promote tumor cell survival and enable cancer progression.

In conclusion, the functional status of autophagy is intricately connected to intracellular ROS levels in complex ways, and these elaborate interactions likely impact tumorigenesis in context-specific manners that are worthy of further investigation and may provide novel insights into how to best pharmacologically modulate autophagy for cancer prevention and treatment.

## Conflict of Interest Statement

The authors declare that the research was conducted in the absence of any commercial or financial relationships that could be construed as a potential conflict of interest.

## Abbreviations

AMBRA: activating molecule in beclin 1-regulated autophagy
AMP: adenosine monophosphate
AMPK: AMP activated protein kinase
ARE: antioxidant response element
ASC: apoptosis-associated speck-like protein containing a CARD
Atg 3: autophagy-related 3
Atg4C: autophagy-related 4C
Atg5: autophagy-related 5
Atg7: autophagy-related 7
Atg8/LC3: autophagy-related 8/microtubule-associated protein light chain 3
Atg10: autophagy-related 10
Atg12: autophagy-related 12
mAtg13: mammalian autophagy-related 13
Atg16L: autophagy-related 16-like
ATM: ataxia-telangiectasia mutated
ATP: adenosine triphosphate
Bcl-2: B cell CLL/lymphoma 2
Beclin1: coiled-coil myosin-like BCL2-interacting protein
BHA: butylated hydroxyanisole
Bif1: Bax interacting factor 1
BNIP3: BCL2/adenovirus E1B 19 kDa interacting protein 3
BNIP3L/Nix: BCL2/adenovirus E1B interacting protein 3-like/NIP3-like protein X
CCCP: carbonyl cyanidem-chlorophenylhydrazone
Deptor: DEP domain containing MTOR-interacting protein
E1: ubiquitin-activating enzyme
E2: ubiquitin-conjugating enzyme
EGF: epidermal growth factor
ER: endoplasmic reticulum
ERK1/2: extracellular regulated kinase 1/2
Ero1: endoplasmic reticulum oxidoreductin-1
ETC: electron transport chain
FGFR-1: fibroblast growth factor receptor-1
FIP200: FAK family kinase-interacting protein of 200 kDa
FOXO1/O3: forkhead box protein O1/O3
GAP: GTPase activating protein
HIF-1α: hypoxia-inducible factor-1 alpha
HMGB1: high mobility group box 1
H_2_O_2_: hydrogen peroxide
iBMKs: immortalized baby mouse kidney cells
IL-1β: interleukin 1 beta
IL-18: interleukin 18
IL-1R: interleukin 1 receptor
iMMECs: immortalized mouse mammary epithelial cells
JNK: c-Jun N-terminal kinase 1
Keap1: Kelch-like ECH-associated protein 1
LKB1: liver kinase B1
MAPK: mitogen-activated protein kinase
MEFs: mouse embryonicfibroblasts
MFN1/2: mitofuscin 1/2
mLST8: MTOR-associated protein,LST8 homolog
MMTV: mouse mammary tumor virus
Mn-SOD: manganese superoxide dismutase
mTORC1: mammalian target of rapamycin 1
NAC: *N*-acetyl cysteine
NADPH oxidase: nicotinamide adenine dinucleotide phosphate-oxidase
NF-κB: nuclear factor kappa B
NLR: Nod-like receptor
NLRP3: NLR family, pyrin domain containing 3
NO: nitric oxide
Nrf2: nuclear factor erythroid 2-related factor 2
$O_{2}^{\cdot -}$: superoxide anion
OH^.^: hydroxyl radical
ONOO: peroxynitrite
p62/SQSTM1: sequestosome 1
p150: phosphatidylinositol 3-kinase-associated p150
PAO: phenylarsine oxide
Parkin: Parkinson protein, E3 ubiquitin protein ligase
PDGF: platelet-derived growth factor
PDK1: phosphoinositide-dependent kinase 1
PE: phosphatidyl ethanolamine
PERK: PRKR-like endoplasmic reticulum kinase
PH: Pleckstrin homology
PI3K/Vps34: phosphatidylinositol 3-kinase/vacuolar protein sorting 34
PINK1: PTEN-induced putative kinase 1
PIP_2_: phosphatidylinositol-4,5-phosphate
PIP_3_: phosphatidylinositol-3,4,5-phosphate
PKB/Akt: protein kinase B-alpha
PRAS40: proline-rich AKT substrate 40 kDa
Prdx1/2: peroxiredoxin 1/2
PTEN: phosphatase and tensin homolog
PyMT: polyoma middle T
Rag: Ras-related GTP-binding protein
RAGE: receptor for advanced glycation end products
Raptor: regulatory associated protein of MTOR, complex 1
Rheb: Ras homolog enriched in brain
RNS: reactive nitrogen species
ROS: reactive oxygen species
RUBICON: RUN domain Beclin 1-interacting cysteine-rich-containing protein
Sesns: sestrins
SOD1: copper/zinc superoxide dismutase
SOD2: manganese superoxide dismutase
TLRs: Toll-like receptors
TSC 1/2: tuberous sclerosis 1/2
UBA: ubiquitin-associated
ULK1/2: Unc-51-like kinase 1/2
UVRAG: ultraviolet irradiation resistant-associated gene
XO: xanthine oxidase.
